# Comparison of trends in habitat and resource selection by the Spanish Festoon, *Zerynthia rumina* , and the whole butterfly community in a semiarid Mediterranean ecosystem

**DOI:** 10.1093/jis/14.1.51

**Published:** 2014-01-01

**Authors:** Raúl Ochoa-Hueso, Daniel de la Puente Ranea, José Luis Viejo

**Affiliations:** 1 Department of Zoology. Universidad Autónoma de Madrid. Ciudad Universitaria de Cantoblanco. C/ Darwin 2,28049 Madrid, Spain; 2 Current address: Hawkesbury Institute for the Environment, University of Western Sydney, Locked Bag 1797,Penrith, New South Wales, 2751, Australia

**Keywords:** habitat selection, larval hostplant, nectar

## Abstract

Butterfly community and single species based approaches were taken to establish conservation priorities within a nature reserve in Central Spain. In this study, patch type (sclerophyllous, halophilous, or disturbed), potential herbaceous nectar availability, potential woody plant nectar availability, total nectar availability, and two approximations to plant diversity (herbaceous and woody plant diversity) were evaluated as variables that account for adult butterfly density. Butterfly communities in the reserve, which consist mostly of generalist species, were denser in relatively wet areas dominated by halophilous vegetation. Diversity did not significantly vary between ecologically different transects. Total nectar availability correlated with higher butterfly densities within both undisturbed and disturbed areas, which could be primarily explained by the lack of water typical of semiarid Mediterranean climates, where fresh, nectariferous vegetation is scarce. Woody plants were also found to be important sources of nectar and shelter. In the dryer sclerophyllous sites, adult butterfly density was best explained by herbaceous plant diversity, suggesting better quality of available resources. The endangered specialist *Zerynthia rumina* (L.) (Lepidoptera: Papilionidae) was only present at the sclerophyllous sites. Its density was very low in all sampled transects, excluding one relatively isolated transect with high larval hostplant density. In contrast to the community-based approach, density of *Z. rumina* adults is better explained by the density of its larval hostplant than by nectar availability, a trend previously described for other sedentary species. Management strategies for protecting insect-rich areas should consider the specific ecological requirements of endangered species.

## Introduction

The Spanish Festoon, *Zerynthia rumina* (L.) (Lepidoptera: Paipilionidae), is a specialist butterfly species protected in Spain. Although categorized as a species of least concern by the European Red List ( van Swaay et al. 2010 ), this species is regionally protected in Madrid, Central Spain ( Gómez de Aizpurua et al. 2009 ). Its distribution range comprises the Iberian Peninsula, Southeastern France, and Northern Africa ( Gómez de Aizpúrua et al. 1999 ). *Z. rumina* inhabits rocky, warm, and dry areas, cultivated lands, flowering prairies, and dry-water streams ( Tolman and Lewington 2002 ). In semiarid ecosystems from Central Spain, it is tightly linked to oak formations and is frequently found flying or resting in sunny, south-facing slopes of ker-mes-oak thickets ( de Viedma et al. 1985 ; Gómez de Aizpúrua et al. 1999 ). In Central Spain, it normally emerges from the pupal stage, in which the species can remain for two years, during the month of April ( Gómez de Aizpúrua et al. 2009 ). It is predominantly univoltine and occasionally bivoltine ( Perceval 1991 ), sometimes having a partial second generation ( Arrebola 1990 ). The larval hostplants of this species belong to the genus *Aristolochia* L. (Piperales: Aristolochiaceae), with populations having the ability to exploit any available species of this genus ( Tolman and Lewington 2002 ). Jordano and Gomariz (1994) discovered a clear link between adult *Z. rumina* density and the nutritional quality of *Aristolochia* spp., with rhizomatous species having higher nutritional quality. At the site of this study in Central Spain, its only larval hostplant is the geophyte *A. pistolochia* L. ( Gómez de Aizpúrua et al. 2009 ).

Most invertebrates included in existing conservation lists are recommended by specialists after an evaluation process ( Lobo 2003 ). It is necessary to support these decisions with first-hand field work in order to ensure effective management practices ( Carrión and Munguira 2001 ). Conservation strategies are most efficient when habitat requirements for a protected species are known. Strong associations between plant communities and butterfly fauna have been documented in the Mediterranean basin ( Baz 1986 ; Viejo and Templado 1986 ; García Barros et al. 1998 ; Tobar et al. 2002; Jimenez-Valverde et al. 2004). This association is stronger for larvae, which are typically more stenophagous ( Viejo and Templado 1986 ) and have reduced mobility compared to adults. It has also been shown that adults of several species select habitat patches with greater availability of nectar and/or larval hostplants ( Brommer and Fred 1999 ; Haddad and Baum 1999 ; Merckx and Van Dyck 2002 ; Scheider et al. 2003; Auckland et al. 2004). Janz et al. (2005) showed that nectar availability can also influence the selection of ovipositing sites in the polyphagous species *Polyommatus icarus* . Adults of some nectar-feeding generalist species have the ability to feed on various sources during the flight period and between years, depending on availability ( Sharp et al. 1974 ; Munguira et al. 1997 ). However, a strong selection for particular nectar sources has been observed in some specialist species ( Bhuyan et al. 2005 ). Different flower visitation patterns have also been observed between sexes, a trend which is not affected by general availability and abundance but related to the chemical composition of nectar ( Rusterholz and Erhardt 2000 ), which suggests different nutritional requirements for males and females. Consequently, the number of plant species used by a certain butterfly population represents its niche width ( Singer 1983 ).

A resource-based concept of habitat can be defined as the intersection and union of the mapped resource locations used by each stage of an organism life cycle ( Dennis et al. 2003 ). In the case of butterflies, habitats would at least include resources for mate location, roosting, thermoregulation, predator escape, and plant-related resources for egg laying or larval and adult feeding. Thus, having previously defined a species’ habitat preferences, it is possible to identify potentially favorable areas for the species. The use of nectariferous and larval-host plant species in restoration practices has been suggested ( Ravenscroft 1994 ; Schultz and Dlugosch 1999 ; Rusterholz and Erhardt 2000 ), especially for conservation of specialist species ( Tooker et al. 2002 ).

Based on literature and field observations, it is hypothesized that *Z. rumina* is not homogeneously distributed across the landscape. Being a monophagous and sedentary species, *Z. rumina* is likely to be associated with patches with high larval hostplant abundance. These areas are dominated by kermes-oak formations, where rosemary ( *Rosmarinus officinalis* L.) and, to a lesser extent, other woody plants such as *Rhamnus lycioides* L., *Lithodora fru-ticosa* (L.) Griseb., or *Retama sphaerocarpa* L. are important sources of nectar and shelter. A second hypothesis is that, given the obvious water limitation in these ecosystems, humid areas such as gullies and small creeks are important conservation areas, offering fresh, nectariferous vegetation to butterfly communities.

Therefore, the aims of this study were (1) to identify habitat characteristics of areas occupied by the specialist butterfly *Z. rumina* ; (2) to assess the relative importance of nectar availability and larval hostplant abundance in *Z. rumina* habitat selection; and (3) to compare patterns of *Z. rumina* habitat selection with that of the whole butterfly community.

## Materials and Methods

The study site was located in the Nature Reserve El Regajal-Mar de Ontígola (Madrid, Spain), a 570-ha area mainly protected to ensure the conservation of its unique butterfly fauna. Within its borders, a total of 73 butterfly species (33% of the 224 diurnal butterfly species present in the Peninsular territory) have been cited, some of which are scarce and unique ( Gómez de Aizpúrua et al. 2009 ). The Reserve is located near Aranjuez (40º 00’ N, 3º 36’ W), between the Jarama and Tajo val-leys, about 45 km south of the city of Madrid, at an altitude of 500–600 m a.s.l. Kermes-oaks dominate the hilltops separated by gullies, and there are also important croplands (mainly vineyards and olive tree fields). Water and washed salts accumulate in the low parts of the hills, areas dominated by halophilous vegetation ( Costa el al. 2005 ). The slopes are usually covered by a welldeveloped biological soil crust and gypso-phytes ( de Viedma et al. 1985 ). The climate is semiarid Mediterranean, with cold winters and hot summers ( Rivas-Martínez 1987 ). The mean annual rainfall is 425 mm yr ^-1^ , mostly concentrated between October and May, with a prolonged summer drought strongly determining the presence of plant and animal communities (Gonzalez Granados 1999).

From 31 March 2006 to 1 May 2006, a total of seventeen 200 m transects (five in disturbed areas, six within patches dominated by halophilous vegetation, and six within sclerophyllous areas) were regularly surveyed following the line transect method ( Pollard and Yates 1993 ). According to this methodology, all butterflies seen 5 meters ahead and 2.5 m at each side of the surveyor were registered during each visit. Given the intrinsic limitations of this methodology in Mediterra-nean-type ecosystems ( Baz and García-Boyero 1995 ), species similar in appearance, almost undistinguishable when flying, were grouped and analyzed together. Several individuals of each identified species were netted in order to be sure of visual identification. Each 200 m transect was surveyed two times each visit for six minutes in opposite directions (a total of 400 m walked in 12 minutes) in order to maximize the probability of detecting non-abundant species. A total of 118 visits were made during the two month sampling period. The relative abundance of a given species in each transect during a single visit resulted from averaging the abundances recorded in each direction. The relative abundance of a species in a given transect for the whole sampling period resulted from averaging the total visit-per-transect data. Surveys were conducted in mild weather conditions, avoiding non-favorable days for flying (rainy or cloudy days, low temperatures, and strong winds) in order to render data collected in different transects and at different times susceptible to statistical treatment ( Brown and Boyce 1998 ). Cloudiness, shade temperature, and wind speed (Beaufort scale) were recorded during each visit in order to ensure similar weather conditions throughout the sampling period.

Seventeen independent transects were delineated using existing bibliography and cartography. Each transect corresponded to one of three previously defined major vegetation formations or patch types in the reserve (Gomez de Aizpurua et al. 2003). Six transects were located within disturbed areas and croplands; six within patches dominated by sclerophyllous vegetation (kermes-oak thickets and Mediterranean dry shrub-lands); and six within halophilous vegetation patches (wet areas with high water table level or close to small creeks, where washed salts accumulate). Transects were visited once a week and always in random order to ensure all locations were surveyed at different times within a six-hour time frame from 10:00 to 16:00 .

During each sampling event, vegetation surveys were conducted prior to adult butterfly estimations to avoid bias. The following biotic indicators related to the extant vegetation were measured: (1) annual, woody plant, and total nectar availability; (2) *A. pistoloquia* abundance; and (3) plant diversity by the use of the Shannon-Wiener Index ( Fowler et al. 1998 ). Nectar availability was estimated at two different scales. First, nectar provided by annual plants and woody plants, as well as total nectar availability, were estimated for each transect by dividing it into four 50 m segments (50 x 5 m ^2^ areas), where availability values were given following a scale of flower density similar to that used by Sharp et al. (1974) : 0 (almost imperceptible), 1 (scarce), 2 (frequent), 3 (abundant), and 4 (very abundant). Transect values for each visit were obtained by summing the four 50 m segment values. The second estimation of nectar availability was made by selecting three spots at the beginning (0 m), middle (100 m), and end (200 m) of each transect in order to have nectar estimates within a 25 m radius perimeter (approximately 393 m ^2^ ) from each spot. The purpose of this method was to account for the peripheral vegetation surrounding transects.

Plant diversity was assessed by placing seven 5 x 0.5 m ^2^ quadrats at regular 25 m intervals across the transects. The Braun-Blanquet scale was used to assign percentage cover of potentially nectariferous herbaceous and woody species (+: presence; 1: 1–5%; 2: 6–25%; 3: 26–50%; 4: 51–75%; 5: 76–100%). The final cover for each species in all transects resulted from summing the averaged values from each assigned interval and dividing by the number of sampling points. Those species not included in any sampling quadrat but present in transects were registered as present (+). This method also allowed quantification of *A. pistolochia* and other larval hostplant species availability. For practical reasons, surveys of vegetation diversity and *A. pistolochia* abundance were only conducted at sclerophyllous transects, the potential habitat for *Z. rumina* at the reserve.

Prior to statistical analysis, data sets were checked for homogeneity in weather conditions. When normality assumptions were not met, data were log-transformed in order to meet parametric tests assumptions or correspondent non-parametric tests were used. Generalized linear models were used to compare butterfly indicators or nectar availability between patch type or preservation status (disturbed vs. undisturbed; sclerophyllous and halophilous). HSD post-hoc Tukey’s test was used for multiple comparisons. Linear regressions were used for relationships between butterfly variables and environmental predictors. When appropriate, other regression models were used. All statistical analyses were conducted using SPSS 17 (IBM, www.ibm.com ). Data is presented untrans-formed when not specified. Significance level was established at *P* = 0.05. Averaged whole-season data from each transect was analyzed.

## Results

### Nectar availability

A positive relationship was found between the availability of nectar within the 200 x 5 m ^2^ transect areas and the availability in the peripheral 25 m radius areas for all three nectar categories: total (woody and herbaceous, *R ^2^* = 0.880, *P* < 0.001), woody plant ( *R ^2^* = 0.781, *P* < 0.001), and herbaceous plant ( *R ^2^* = 0.880, *P* < 0.001). Total and herbaceous nectar values were significantly related ( *R ^2^* = 0.987, *P* < 0.001), whereas total nectar availability was not related to woody plant nectar availability ( *R ^2^* = 0.159, *P* = 0.222). Woody plant nectar availability was higher in the undisturbed patches (ANOVA; *F* = 5.071, *P* = 0.040). Total and herbaceous nectar availability mean values did not significantly differ between habitats.

### Butterfly community pattern

**Figure 1.**
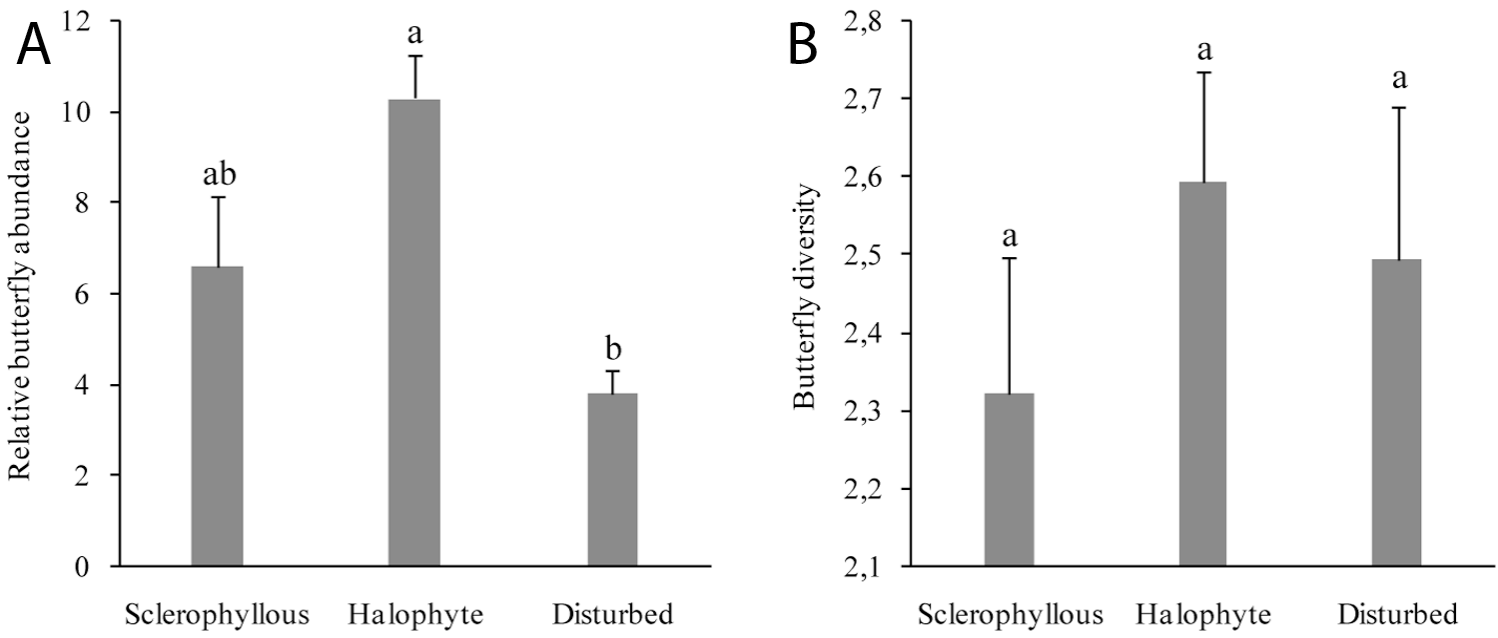
Relative butterfly abundance (a) and butterfly diversity (b) with respect to three major plant stand types. Different lowercase letter above SE bars indicates significant differences. Sclerophyllous and halophytes sites, *N* = 6; disturbed sites, *N* = 5. High quality figures are available online.

Nineteen butterfly species were detected in the surveys ( Table 1 ). Only total butterfly density values, except for *Z. rumina* density values, were analyzed in this paper. Mean density of adult butterflies significantly varied with regard to patch type (ANOVA; *F* = 8.892, *P* = 0.003; Figure 1a ). The highest density was found in the halophilous transects, and significantly lower values were found in disturbed patches. Sclerophyllous transects had intermediate butterfly abundances. Butterfly diversity was not different with regard to patch type (ANOVA, *F* = 0.694; *P* = 0.516; Figure 1b ).

**Table 1.**
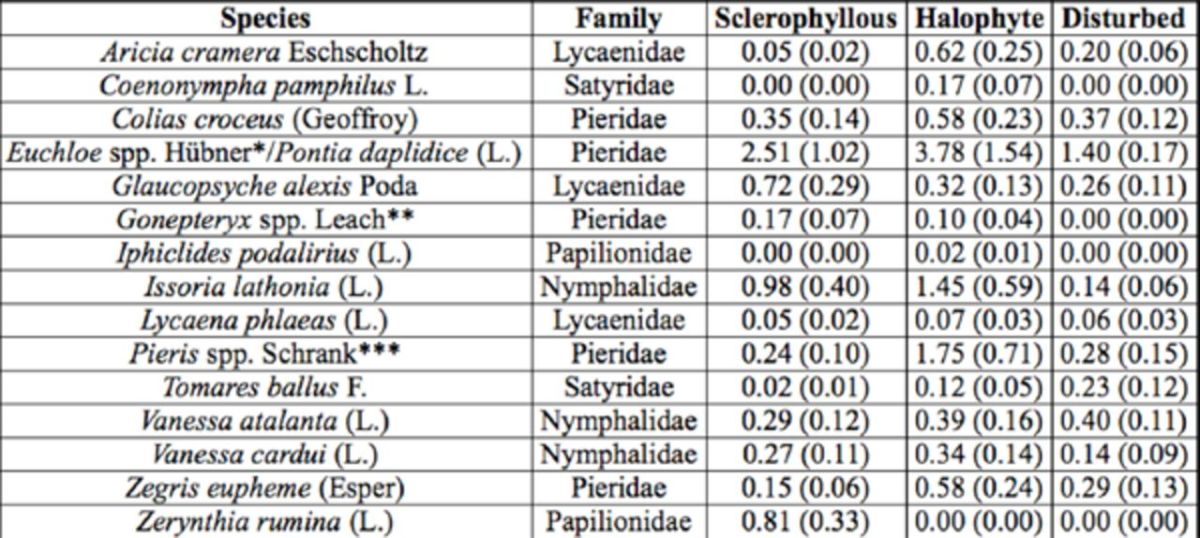
List of the nineteen butterfly species found during the surveys. Average adult density per transect and standard error (within parenthesis) are shown for each vegetation type

* *E. ausonia* ; *E. tagis*

** *G. cleopatra* ; *G. rhamni*

*** *P. brassicae* ; *P. rapae*

**Figure 2.**
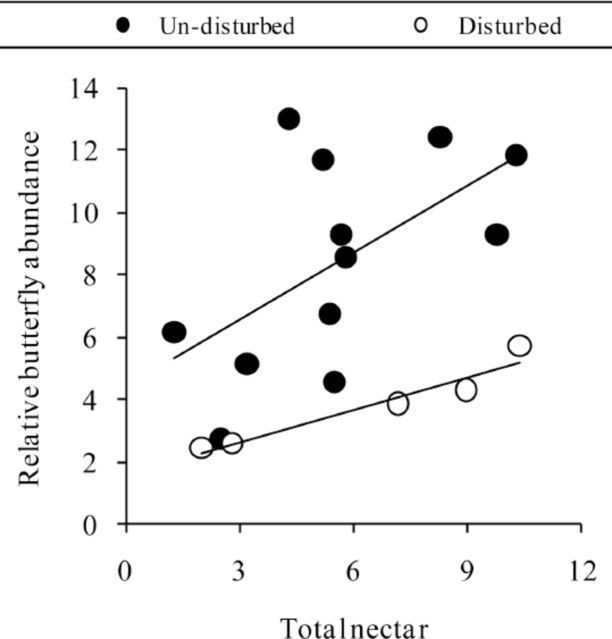
Relationship between potential nectar availability and relative butterfly abundance within undisturbed and disturbed sites. Undisturbed sites, *N* = 12; disturbed sites, *N* = 5. Regressions lines are shown when significant (see results section). High quality figures are available online.

Butterfly abundance was best, although weakly, explained by woody plant nectar availability ( *R ^2^* = 0.155; *P* = 0.118) when ecological differences between patches were not taken into account. However, when undisturbed and disturbed sites were analyzed separately, strong and significant positive relationships were found between total butterfly density and both herbaceous (undisturbed sites: *R ^2^* = 0.393, *P* = 0.029; disturbed sites: *R ^2^* = 0.929, *P* = 0.008) and total (undisturbed sites: *R ^2^* = 0.336, *P* = 0.048; disturbed sites: *R ^2^* = 0.929, *P* = 0.008; Figure 2 ) nectar availability . Conversely, woody plant nectar availability was not related to butterfly abundance in undisturbed or disturbed areas when analyzed separately. Butterfly diversity was not related to any of the habitat quality indicators, even when analyses were carried out separately for patch type or plant cover preservation status. When analyzed separately, each butterfly species showed a different pattern in relation to nectar availability (only *Z. rumina* shown in Figures).

**Figure 3.**
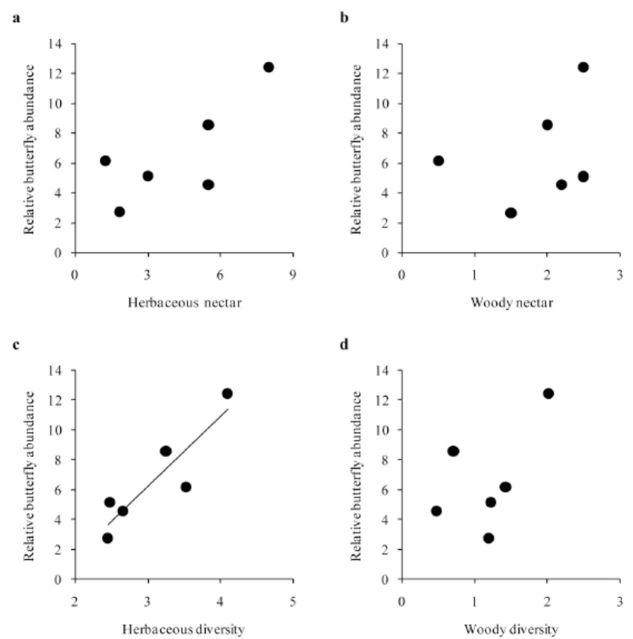
Relationship between potential herbaceous (a) or woody (b) nectar availability and herbaceous (c) or woody (d) plant diversity with relative butterfly abundance within sclerophyllous sites. *N* = 6. Regressions lines are shown when significant (see results section). High quality figures are available online

In sclerophyllous sites, butterfly density was positively related to herbaceous plant diversity ( *R ^2^* = 0.800, *P* = 0.015; Figure 3c ) but not to woody plant diversity ( *R ^2^* = 0.300, *P* = 0.261; Figure 3d ) or total nectar ( *P* > 0.05; Figure 3a , b ). Butterfly diversity was also unrelated to herbaceous ( *R ^2^* = 0.004, *P* = 0.904) and woody ( *R ^2^* = 0.043, *P* = 0.696) plant diversity.

***Zerynthia rumina***

**Figure 4.**
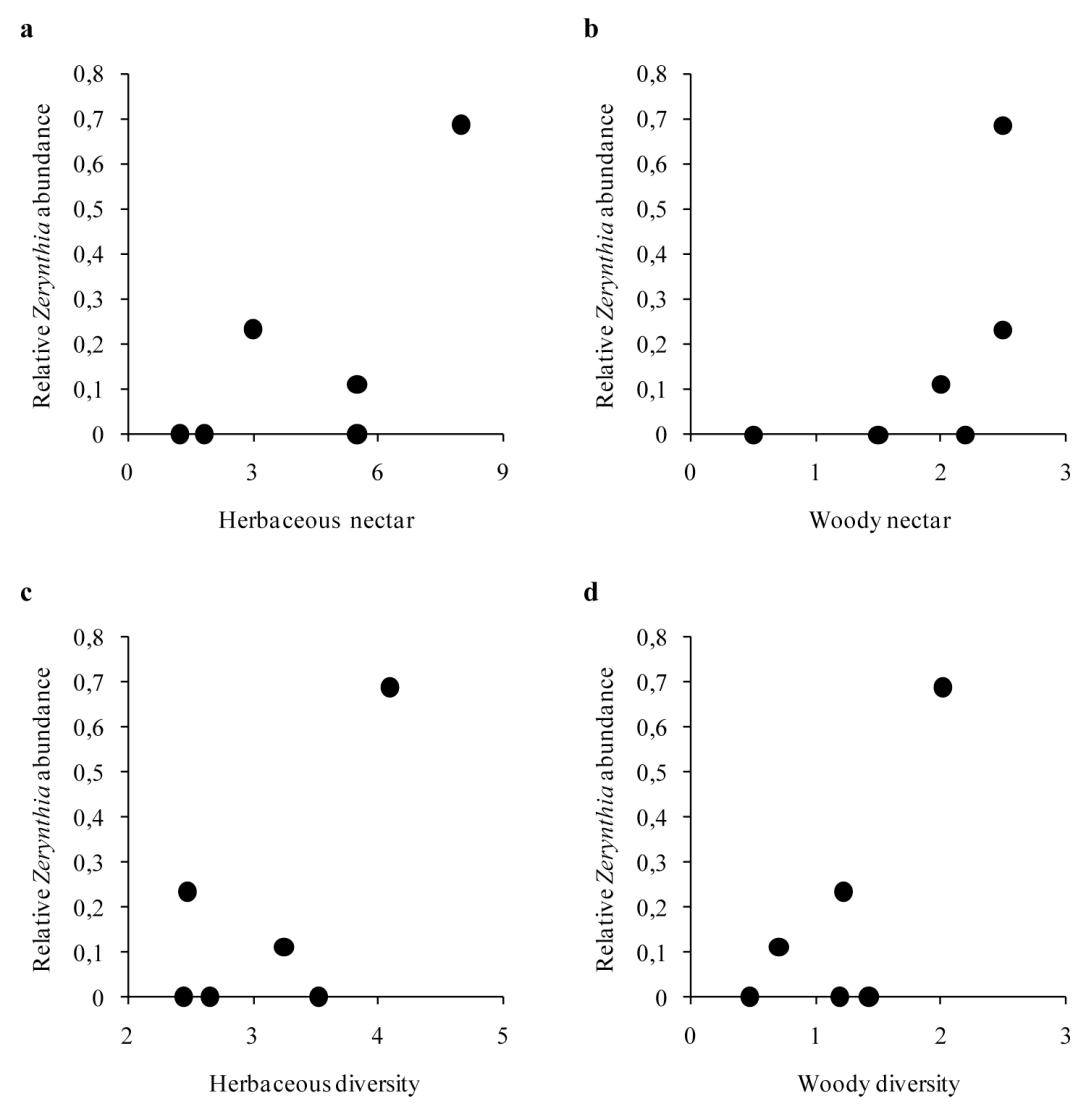
Relationship between potential herbaceous (a) or woody (b) nectar availability and herbaceous (c) or woody (d) plant diversity with relative *Zerynthia rumina* abundance (represented as log10-scale) within sclerophyllous sites. *N* = 6. Regressions lines are shown if significant (see results section). High quality figures are available online.

**Figure 5.**
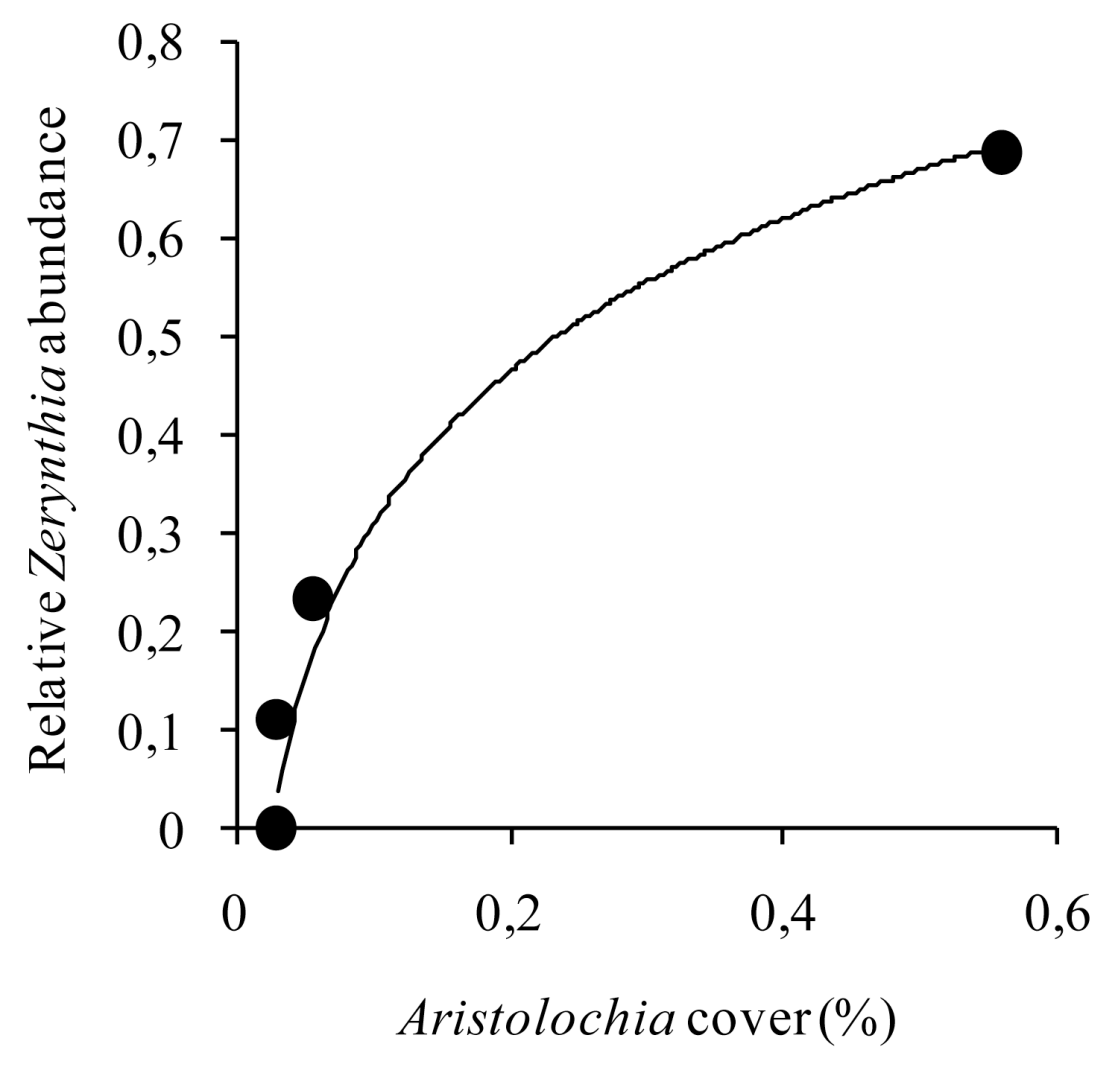
Logarithmic regression between *Zerynthia rumina* and its larval hostplant within sclerophyllous sites. *Z. rumina* and *Aristolochia pistolochia* are both in log10-scale. *N* = 6. See results section for further details. High quality figures are available online.

Sclerophyllous areas (rosemary shrub-lands) were selected by *Z. rumina* (Kruskall-Wallis, χ ^2^ = 6.216, *P* = 0.045), and the species was not present in any of the halophilous or disturbed transects. Adult density was not related to total ( *R ^2^* = 0.531, *P* = 0.100) or herbaceous ( *R ^2^* = 0.513, *P* = 0.109; Fig. 4a ) nectar availability. Although butterfly density was not related to woody plant nectar availability ( *R ^2^* = 0.336, *P* = 0.227) there appeared to be a threshold for woody plant nectar availability above which butterfly densities increased at a high rate ( Figure 4b ). Adult density was also significantly explained by larval hostplant abundance (log-regression, *R ^2^* = 0.967, *P* < 0.001; Figure 5 ). Herbaceous ( *R ^2^* = 0.424, *P* = 0.161; Figure 4c ) and woody plant diversities ( *R ^2^* = 0.541, *P* = 0.096; Figure 4d ) did not explain adult density.

## Discussion

Nectar sources seemed to be homogenously distributed at a small scale (within transect and peripheral areas), indicated by the strong relationship between within-transect and outside-transect nectar availability values in the patches. This result supports the use of transects as representatives of their immediate surroundings. Due to the general scarcity of woody plants at a larger scale, especially in disturbed areas, most of the potentially available nectar in the reserve was being provided by more abundant and widespread herbaceous plants. In contrast, woody plant nectar was not homogeneously distributed, being present only in well-preserved patches.

High total nectar availability correlated with higher butterfly densities in both undisturbed and disturbed areas, which suggests a preference for patches rich in nectar sources for adults. It is noteworthy that observed butterfly density values increased more rapidly in undisturbed sites as nectar availability increased. This pattern could be explained by the absence of woody plant cover, which makes herbaceous plants the major nectar source for adults in these areas. Most of the analyzed taxa were polyphagous and generalist species, known to select nectar sources depending on availability rather than nutritional quality ( Munguira et al. 1997 ). This could explain the strong relationship found between butterfly abundance and herbaceous nectar availability. It is possible that during the spring months, widespread herbaceous nectar is selected following quantity-based criteria while woody plant nectar, more scarce and present only in certain areas, is selected by adults based on the nectar’s nutritional value.

The highest butterfly densities were found within halophilous transects rich in woody vegetation. High woody-plant–nectar availability within the well-preserved areas would cause a positive effect on butterfly density. This effect is less likely in disturbed areas where woody plants are scarce or completely absent. Woody-plant nectar could play a key role in increasing adult fitness by providing essential amino acids and sugars ( Mevi-Schütz and Erhardt 2005 ), even though nectar production is not the rule for woody plants in water-stressed Mediterranean systems (Herrera 1985). Although higher nectar availabilitywas assumed when flower density was higher, nectar production and composition can be altered depending on plant physiological status ( Rusterholz and Erhardt 2000 ). Additionally, two main nectar types, those rich in sucrose and those rich in hexose, have been described ( Dupont et al. 2004 ). Shrubs could also be regarded as more reliable resources because of their ability to exploit not only seasonal water provided by spring rains but also deeper water table. This is especially important during the dry and hot summer months, when annual plants are no longer available. This lead us to hypothesize that the water contained in flower nectar, not just its sugar or amino acid content, could be the resource primarily selected for by generalist species in water-limited are as. Viejo (1982) provided evidence of seasonal butterfly micro-migrations from dryer to wetter areas within the nature reserve, which supports this view and is in accordance with the fact that halophilous patches hosted higher adult butterfly numbers. Further investigation is required to determine the nutritional importance of different nectar sources and to provide reliable nectar production data for common shrubs in water-limited Mediterranean-type ecosystems.

The observed preference for halophilous vegetation shown by butterflies could also be due to the humid microclimate provided by woody vegetation, rather than a preference for woody plant nectar sources. Jiménez-Valverde et al. (2004) suggested that the key role played by woody plants in Mediterranean ecosystems is providing shelter against strong winds and protection from direct solar radiation during the summer drought. Thus, the low adult butterfly abundances observed in disturbed patches could be related to the absence of woody vegetation. Butterfly conservation strategies should preserve the quality of areas with high water availability in semiarid Mediterranean ecosystems ( Viejo et al. 1985 , 1992).

*Z. rumina* density was observed to be higher in the sclerophyllous transects, which is in agreement with our initial hypothesis. However, adult density of this species was strikingly low even in its preferred habitat, sometimes being completely absent from surveyed sclerophyllous transects. In addition, some individuals were captured during the 2005 flying season at certain sites where it seemed to be absent in 2006 (de la Puente, unpublished data). We attribute this absence to the effects of the extremely severe drought of 2005, which could have significantly reduced the water availability for plants. *A. pistolochia* did not sprout in 2005 (Gomez de Aizpurua et al. 2009). This reduced larval-hostplant availability could have increased larval mortality, explaining the low numbers of adults emerged from pupae observed during the following 2006 flying season. This highlights the importance of water availability for maintaining healthy *Z. rumina* populations.

Being a monophagous butterfly, *Z. rumina* adult distribution is conditioned by larval hostplant presence and abundance. In addition, monophagous larvae have been related to short flying distances by adults from patch to patch ( Scott 1975 ), which would explain the complete absence of *Z. rumina* from patches without *Aristolochia* . *Z. rumina* ’s early emergence, short flying period, and sedentary behavior make it less likely to select habitats for their nectar availability (Gomez de Aizpurua et al. 2009). Therefore, larval-hostplant micro-distribution stands as the main factor explaining *Z. rumina* habitat selection in the reserve. Adult feeding resources would consequently play a secondary role, although these should be also present in the patch. These results also support protecting areas with high *Aristolochia* density as the main strategy for preserving healthy *Z. rumina* populations.

The transect showing the highest abundance of *Z. rumina* adults is located in a patch relatively isolated from other potentially suitable habitats by a busy, 200 m wide highway, vineyards, olive tree fields, and a stream covered by tall halophilous vegetation on both banks. With the purpose of investigating the mobility of *Z. rumina* , one of the banks was also surveyed every time the transect was visited during the campaign. No *Z. rumina* individuals were ever detected along these banks, while most of the other species registered 50 m away in the transect were present at the stream (de la Puente, unpublished data). This fact suggests low mobility and dispersion abilities of *Z. rumina* in the reserve and could also explain the high dependence on larval hostplant abundance. This would make the species enormously dependent on weather conditions. It also stresses an extremely important influence of habitat fragmentation on population connectivity and genetic flux. The patch mentioned above could act as a source, maintaining the viability of smaller and weak-er surrounding sink populations. During the spring of 2008, a mark-recapture study of this species at the same study site gave a total number of less than 30 individuals (de la Puente, unpublished data). Having already been effected by severe droughts, this small, isolated *Z. rumina* population could go locally extinct by capricious stochastic reasons such as low female abundance. Considering *Z. rumina* ’s low dispersal ability, the effects of fragmentation could be devastating for this species, preventing recolonization of dwindling populations.

Monophagous and sedentary butterfly species, being the most threatened, will require increased attention in the future by conservation biologists ( Boggs and Murphy 1997 ). Although woody-plant diversity has been considered a reliable indicator of butterfly communities, our study shows a very different ecological pattern in one of the most endangered species within the community. This highlights a need for considering each species’ particular requirements and ecology. The results indicate that sole consideration of the priorities of butterfly communities could lead to imperfect management, especially when generalist species are more abundant. Singular conditions and conservation statuses of locally endangered species should be carefully considered in order to implement effective management and restoration practices.
